# Forest canopy closure estimation in mountainous southwest China using multi-source remote sensing data

**DOI:** 10.3389/fpls.2025.1629146

**Published:** 2025-08-12

**Authors:** Wenwu Zhou, Qingtai Shu, Cuifen Xia, Li Xu, Qin Xiang, Lianjin Fu, Zhengdao Yang, Shuwei Wang

**Affiliations:** ^1^ Guangyuan Forestry Workstation, Guangyuan, China; ^2^ College of Forestry, Southwest Forestry University, Kunming, China; ^3^ Faculty of College of Soil and Water Conservation, Southwest Forestry University, Kunming, China

**Keywords:** ICESat-2/ATLAS, Bayesian optimization algorithm, machine learning method, geographically weighted regression, multi-source remote sensing data, forest canopy closure

## Abstract

Forest canopy closure (FCC) is an important biological parameter to evaluate forest resources and biodiversity, and the use of multi-source remote sensing synergy to achieve high-accuracy estimate regional FCC at low cost is a current research hotspot. In this study, Shangri-La City, a mountainous area in southwest China, was considered as the research area. The satellite-borne LiDAR ICESat-2/ATLAS data were used as the main information source. Combined with 54 measured plot data, the improved machine learning model of the Bayesian optimization (BO) algorithm was used to obtain the FCC in the footprint-scale ATLAS footprint. Then, the multi-source remote sensing image Sentinel-1/2 and terrain factors were combined to perform regional-scale FCC remote sensing estimation based on the geographically weighted regression (GWR) model. The research results showed that (1) among the 50 extracted ATLAS LiDAR feature indices, the best footprint-scale modeling factors are Landsat_perc, h_dif_canopy, asr, h_min_canopy, toc_roughness, and n_touc_photons after random forest (RF) feature variable optimization; (2) among the BO-RFR, BO-KNN, and BO-GBRT models developed at the footprint scale, the FCC results estimated by the BO-GBRT model were the best (*R* ^2^ = 0.65, RMSE = 0.10, RS = 0.079, and *P* = 79.2%), which was used as the FCC estimation model for 74,808 footprints in the study area; (3) taking the FCC value of ATLAS footprint scale in forest land as the training sample data of the regional-scale GWR model, the model accuracy was *R* ^2^ = 0.70, RMSE = 0.06, and *P* = 88.27%; and (4) the *R*² between the FCC estimates from regional-scale remote sensing and the measured values is 0.70, with a correlation coefficient of 0.784, indicating strong agreement. Additionally, the average FCC is 0.50, predominantly distributed between 0.3 and 0.6, comprising 68.43%. These findings highlight the advantages of mountain FCC estimation using ICESat-2/ATLAS high-density, high-precision footprints and the fact that small-sample estimation results at the footprint scale can serve as training data for the regional-scale GWR model, offering a reference for low-cost, high-precision FCC estimation from footprint scale to regional scale.

## 1 Introduction

Forest canopy closure (FCC) refers to the ratio of crown projection area to forest area in the stand (Meng, 2006) and is a basic parameter of stand structure and stand environment, as well as an evaluation index of forest tending and cutting (Meng, 2006; Chen et al., 2019; Li and Mao, 2020; Hua and Zhao, 2021; Pu et al., 2021); timely and non-destructive high-precision estimation of FCC is of great significance for understanding and monitoring the impact of human activities and climate change on forest ecosystems (Wang et al., 2015). The traditional direct measurement methods of FCC mainly include canopy closure measuring instrument, crown projection, measuring line, and artificial visual (Meng, 2006; Wang et al., 2015), which rely on manual and small-scale accurate measurement, but these are time-consuming, labor-intensive, and ineffective (Chen et al., 2019); furthermore, they cannot meet the research standards of the spatial distribution and variation of FCC at large spatial scales (Wang et al., 2015). The development and application of remote sensing technology combined with small sample standard measured data for regional-scale FCC inversion is a common method due to the low-cost, high-efficiency, and global coverage of remote sensing data resources (Lee and Lucas, 2007; Yang et al., 2022).

At present, there are many studies on estimating FCC using optical remote sensing data or airborne light detection and ranging (LiDAR) data combined with different methods (Lee and Lucas, 2007; Hua and Zhao, 2021; Xu et al., 2022; Yang et al., 2022; Duan et al., 2023). However, optical remote sensing data are susceptible to spectral saturation, and airborne LiDAR data are expensive and difficult to obtain. Using synthetic aperture radar (SAR) data (Neumann et al., 2009; Varghese and Joshi, 2015) and spaceborne LiDAR [ice, cloud, and land elevation satellite/geoscience laser altimeter system (ICESat/GLAS)] data to estimate FCC is relatively inadequate (Wang et al., 2015; Cui et al., 2021), but the GLAS footprint is larger (footprint diameter is 70 m and footprint interval is 170 m), and it is susceptible to terrain, especially in complex alpine regions, which will lead to an improvement in FCC estimation accuracy. Compared with ICESat-2/ATLAS (ice, cloud, and land elevation satellite/advanced topographic laser altimeter system), ATLAS has a smaller footprint size (footprint diameter is only 17 m and footprint interval is 0.7 m), which greatly reduces the influence of terrain on spot echo (Moudrý et al., 2022). In the studies conducted by Hua and Zhao (2021); Li and Mao (2020), and Yang et al. (2022), data from over 70 measured samples combined with remote sensing data were used to quantitatively invert the FCC. However, this study investigated only 54 samples, which not only meets the principle of a large sample size (50) and the accuracy requirements of field investigations (Song et al., 2022a), but also reduces experimental costs. Currently, numerous studies focus on the inversion of forest vertical structure (e.g., forest canopy height and forest height), forest biomass, and understory topography using the latest generation of photon-counting spaceborne LiDAR and ICESat-2/ATLAS (Narine et al., 2019; Lin et al., 2020; Zhu et al., 2020; Song et al., 2022a, b). However, there are few studies focusing on the estimation of forest horizontal structure parameters (e.g., leaf area index and FCC) (Xi et al., 2023). Because the ATLAS footprint data show a spatial discontinuous distribution in the form of strips, it cannot meet the requirements for full coverage in the study area (Narine et al., 2019; Zhu et al., 2020). Therefore, the parameter indicators need to be predicted by choosing the spatial interpolation method or spatial regression method in geostatistics in order to obtain the faceted attribute data covering the continuity of the whole study area and then realize the remote sensing mapping of the FCC (Wang et al., 2015; Zhu et al., 2020; Zhou et al., 2023; Yu et al., 2024; Zhou et al., 2024). For example, it combines ground target information from continuous points with continuous surface remote sensing data (e.g., Sentinel-1/2) to achieve multi-source integration and assimilation of multi-sensor and auxiliary data. This approach aims to improve the accuracy of FCC estimation (Zhao et al., 2016; Zhou et al., 2023, 2024) and enable FCC remote sensing mapping at the regional scale.

ICESat-2/ATLAS and Sentinel-1 are active remote sensing technologies. ATLAS employs advanced photon-counting LiDAR technology, featuring more sensitive single-photon detectors and a higher pulse repetition frequency (Neumann et al., 2019), enabling the acquisition of photon point cloud data with smaller footprints and higher sampling density (Lin et al., 2020). The C-band of SAR possesses penetrability and dielectric properties, making it resilient to factors such as region, time, and climate during imaging, thus capturing the structural characteristics of forests (Zhang et al., 2022). However, it lacks rich spectral information. The multi-spectral sensor of Sentinel-2 can capture electromagnetic radiation information outside the canopy, providing rich canopy data. Its red-edge band enhances FCC estimation accuracy (Hua and Zhao, 2021), but it is limited in acquiring information about tree trunks and branches. Characteristic variables significantly impact the estimation accuracy and inversion results of the model (Zhang et al., 2022). Therefore, in order to reveal the explanatory and contribution of multiple variable factors to FCC, reduce the influence of spectral saturation on vegetation (Zhao et al., 2016), and improve the prediction accuracy of the model, the independent variable factors at the spot scale in this study were determined by 50 parameter values extracted by ICESat-2/ATLAS after feature variable optimization. At the regional scale, the commonly used remote sensing factors such as SAR factor, texture feature, vegetation index and single band reflectivity (Chen et al., 2019; Zhang et al., 2022), and the necessary auxiliary data terrain factor were selected to construct the FCC extrapolation model.

Based on the measured sample size and remote sensing dataset, an appropriate model is selected to achieve the best estimation results (Shu et al., 2022). Machine learning methods offer greater advantages in model fitting accuracy and inversion results compared to traditional statistical methods (Shu et al., 2022). In the canopy closure studies by Hua and Zhao (2021); Wang et al. (2015), and Xu et al. (2022), nonparametric models demonstrated the highest accuracy, and the estimation results were verified. However, the lack of optimization algorithms suggests that model accuracy and estimation results can be further improved. In this study, machine learning methods such as K-NN, RFR, and GBRT are selected as the basic models at the footprint scale. The Bayesian optimization (BO) algorithm is then employed to enhance the performance of these basic models, aiming to construct the optimal FCC estimation model. The BO algorithm leverages prior knowledge to approximate the posterior distribution of the unknown objective function and then selects the next best hyperparameter combination based on this distribution, thereby quickly reducing the computational load while optimizing model performance and improving estimation accuracy (Cui and Yang, 2018; Zhang et al., 2021b). As a sequential optimization method, BO effectively explores and balances the known parameter space and the unknown parameter space through surrogate models and acquisition functions; it is capable of obtaining a globally approximate optimal solution with minimal evaluation costs, thereby avoiding the pitfalls of local optima (Cui and Yang, 2018; Zhang et al., 2021b). In the same nonparametric optimization model, the BO algorithm can reduce simulations for model optimization and improve the model operation rate, increase model estimation accuracy, and provide forecast uncertainty more than particle swarm optimization (PSO), genetic algorithm (GA), and differential evolution (DE) (Zhang et al., 2021b). It is one of the commonly used algorithms to optimize the performance of nonparametric models. This study employed the geographically weighted regression (GWR) model to construct an FCC estimation model for the study area at a regional scale. The GWR model is a local spatial regression technique that predicts unknown spatial variables using known data (Guo et al., 2012; Nazeer and Bilal, 2018; Zhang et al., 2019b; Song et al., 2022c). Although widely used in urban geography, forestry, and other disciplines (Guo et al., 2012; Zhang et al., 2019b), its application to medium-scale and large-scale FCC estimation remains uncommon.

At present, there are few studies on the use of ICESat-2/ATLAS data for estimating FCC, particularly in combination with spaceborne LiDAR and multi-source remote sensing data for cost-effective, regional-scale canopy closure inversion. In this study, ICESat-2/ATLAS data were employed to extract the modeling parameters. Using the BO-RFR, BO-KNN, and BO-GBRT models, the optimal FCC estimation model for footprints was constructed from 54 measured plot datasets. Sentinel-1/2 imagery and digital elevation model (DEM) data were used as sources to extract remote sensing factors. After conducting an OLS (ordinary least squares) test and normal transformation, an FCC extrapolation model was constructed using the GWR model to obtain continuous spatial distribution of FCC information across the study area. The aims of this study are to construct a portable footprint canopy closure estimation model and to explore and verify the feasibility and reliability of the regional FCC estimation method based on the GWR model and multi-source remote sensing data. At the same time, the optimization ability of the BO algorithm to the machine learning model is explored, and a low-cost and high-precision method of estimating FCC is proposed.

## 2 Research materials

### 2.1 Study area

**Figure 1:**
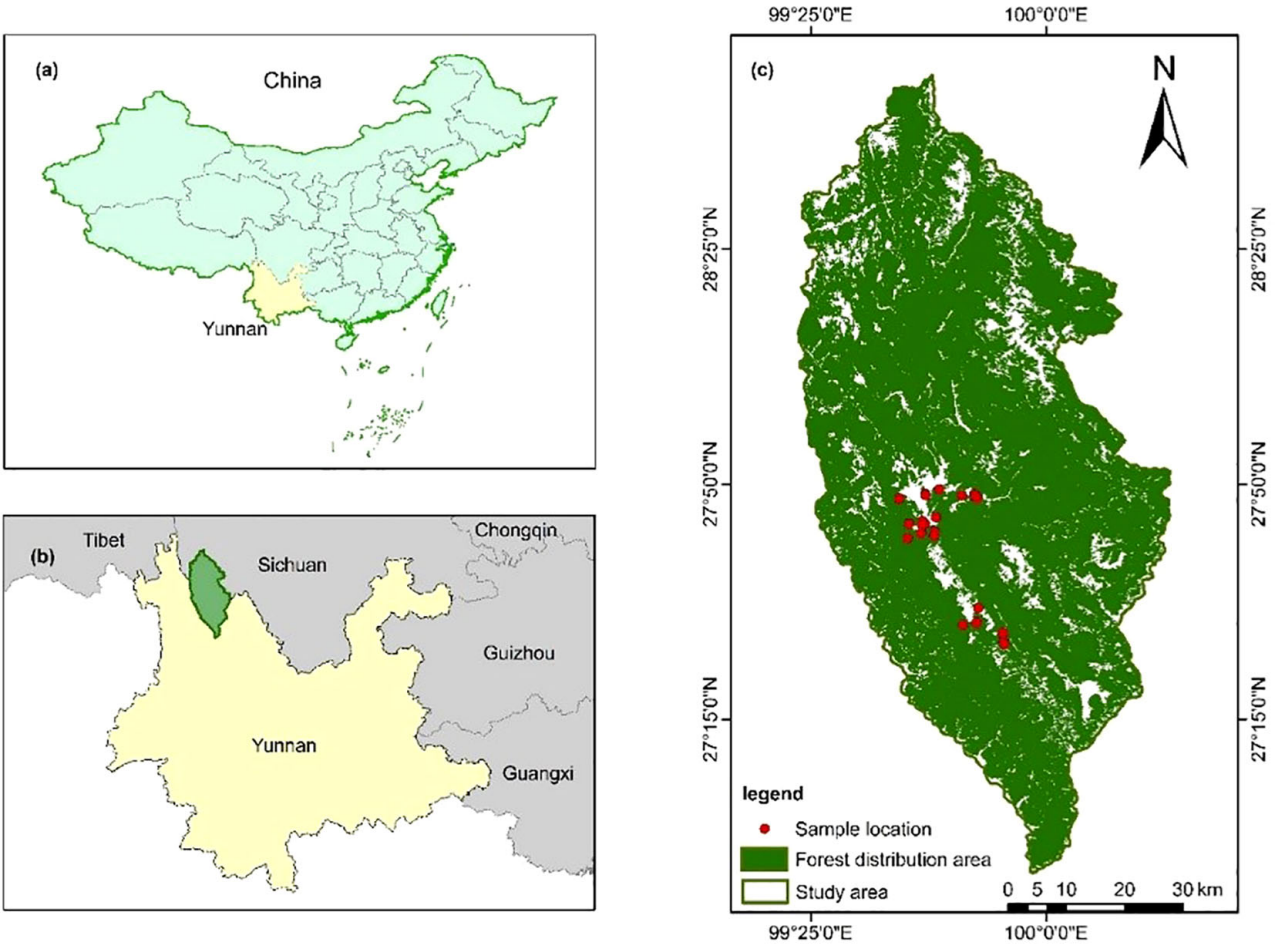
Location of the study area. Shangri-La City in the northwest of Yunnan Province in southwestern China. [**(a)** the study area is located in Southwestern China, **(b)** Shangri-La City is part of Yunnan Province, and **(c)** green is the forest distribution area, red is the 54 sample plots.].

Shangri-La City is located in the northwest of Yunnan Province, China (latitude 26°52′11.44″–28°50′59.57″ N, longitude 99°23′6.08″–100°18′29.15″ E), which belongs to a typical alpine terrain of the Yunnan, Sichuan, and Tibet triangle region (Shu et al., 2022; Song et al., 2022b), as shown in **Figure 1**. The general trend of terrain in the area is high in the northwest and low in the southeast, with a relative elevation difference of 4,042 m; moreover, the average altitude is 3,459 m, the average temperature is 4.7°C–16.5°C, and the average annual rainfall is 649.4 mm; in summary, it belongs to the mountain cold temperate monsoon climate. The total land area of Shangri-La city is 1,141,739 ha^2^, of which forestry land area is 950,911.7 ha^2^, which accounts for 83.3% of the total area; the forest coverage rate reaches 76%, which is an important protection forest area in Yunnan Province of China. Typically, there are 10 vegetation types distributed in the city and the main vegetation type is cold temperate coniferous forests including *Picea asperata*, *Abies fabri*, *Pinus densata*, *Quercus semecarpifolia*, and *Larix gmelinii* (Shu et al., 2022; Song et al., 2022b; Xi et al., 2023).

### 2.2 Sample plot design and data preprocessing

The 54 sample plot data used in the study were sampled in November 2021 in Shangri-La City. The experimental design selects 54 sample circles with a radius of 8.5 m and an area of approximately 0.023 ha^2^, which are consistent with the footprint size emitted by the ATLAS sensor mounted on ICESat-2; it covers the main forest vegetation types at different slopes and altitudes in the study area and records the coordinate information of the samples, tree species, diameter at breast height, tree height, and the measurement of the FCC. Among them, the latitude and longitude coordinates of the center point of the sample circle are consistent with the coordinates of the footprint center of ICESat-2/ATLAS, and using the southern mapping T66 Real-Time Kinematic (RTK) in the fixed solution state, the mean value of five consecutive point lofting was taken by the differential positioning instrument of Thousand Seeker SR3 (Pro version), the error between the center point coordinates of all sample sites and those of the footprints was less than 0.02 m, and, finally, the latitude and longitude coordinates of the center point of the sample circle are determined. In this paper, the measuring line method (Meng, 2006) is used to calculate the FCC of 54 sample circles (**Table 1**). The definition of the measuring line method is as follows: to select a representative section in the sample circle, set up a certain length of the measuring line, along the line to observe the projection of the crown of each tree, and measure the projection length, the crown of the projected length of the sum of the length of the measuring line, and the length of the measuring line for the value of the degree of canopy closure (Meng, 2006).

**Table 1:** Descriptive analysis of FCC statistics.

Statistical	Maximum	Minimum	Mean	SD	Variance	Median
54	0.83	0.20	0.50	0.176	0.031	0.5

### 2.3 ICEsat-2/ATLAS data products acquisition and preprocessing

#### 2.3.1 ICEsat2/ATLAS data acquisition

The ICESat-2 satellite was the first to be equipped with a photon-counting LiDAR payload on a spaceborne platform. In September 2018, it was successfully launched by NASA (National Aeronautics and Space Administration) at the Vandenberg Space Force Base in the United States. The ATLAS system laser on board launched a total of six laser beams at a time. The photon point cloud data with a footprint diameter of 17 m and a sampling interval of 0.7 m were obtained (Neumann et al., 2019; Moudrý et al., 2022); its 22 standard data products are divided into four levels and stored in the US Ice and Snow Data Center (https://nsidc.org/data/icesat-2/data-sets) in HDF5 format (Neumann et al., 2019; Lin et al., 2020; Zhu et al., 2020). The ATL03 global positioning photon data contain six laser beam bands, which are evenly segmented at a distance of 20 m along the track, labeled as gt11–gt3r, and record the time, latitude and longitude of all photon events. Geospatial location information, as well as information such as the number of photons, belongs to the secondary product data. Based on this, the source data can generate more advanced products (Zhu et al., 2020). The ATL08 product is a geophysical data product containing ground elevation information and vegetation height information generated in 100-m segments along the orbital direction based on ATL03 data after further noise removal and signal photon classification (Zhu et al., 2020; Moudrý et al., 2022). This study utilized free ICEsat2/ATLAS data obtained from the earthdata website (https://search.earthdata.nasa.gov/). All ATL03 and ATL08 data products from January 2020 to June 2021 in Shangri-La were selected, and each dataset comprises a total of 118 data points, 354 tracks, and 708 photon trajectory beams.

#### 2.3.2 Photon point cloud denoising and classification algorithm

Because ATLAS is a more sensitive single-photon detector compared to GLAS and has a higher pulse repetition frequency and a weak signal emission, it also captures a significant amount of noise photons when receiving reflected photons from specific ground targets (Neumann et al., 2019; Zhu et al., 2020). Therefore, to use these data for quantitative remote sensing inversion, noise photons must be removed to improve the accuracy of model estimation. In this paper, we employ a combination of the different densities-based spatial clustering of applications with noise (DDBSCAN) and k-nearest neighbors-based (KNNB) algorithms (Zhang et al., 2021a) for denoising. It is demonstrated that this combined approach outperforms the use of either the DDBSCAN or KNNB algorithm alone (Nie et al., 2018; Zhang et al., 2021a). Additionally, the final measurement parameter is replaced by the maximum density difference in DDBSCAN to address the impact of photon density inconsistency on algorithm performance.

The signal photons after denoising need to be accurately classified, which are mainly divided into ground photons and canopy top photons. The classification results will affect the inversion and mapping accuracy of forest parameters (Zhu et al., 2020). The progressive triangular irregular network (TIN) densification (PTD) method was used to distinguish the photon point cloud data into ground photons and canopy photons (Nie et al., 2017, 2018; Zhang et al., 2021a). This method has high ground photon recognition accuracy in complex terrain areas such as large altitude drop. In order to further improve the classification accuracy, the ground point is set to the lowest elevation point under the farthest point from TIN.

#### 2.3.3 Footprint-scale parameter extraction and forest footprint distribution map

**Figure 2:**
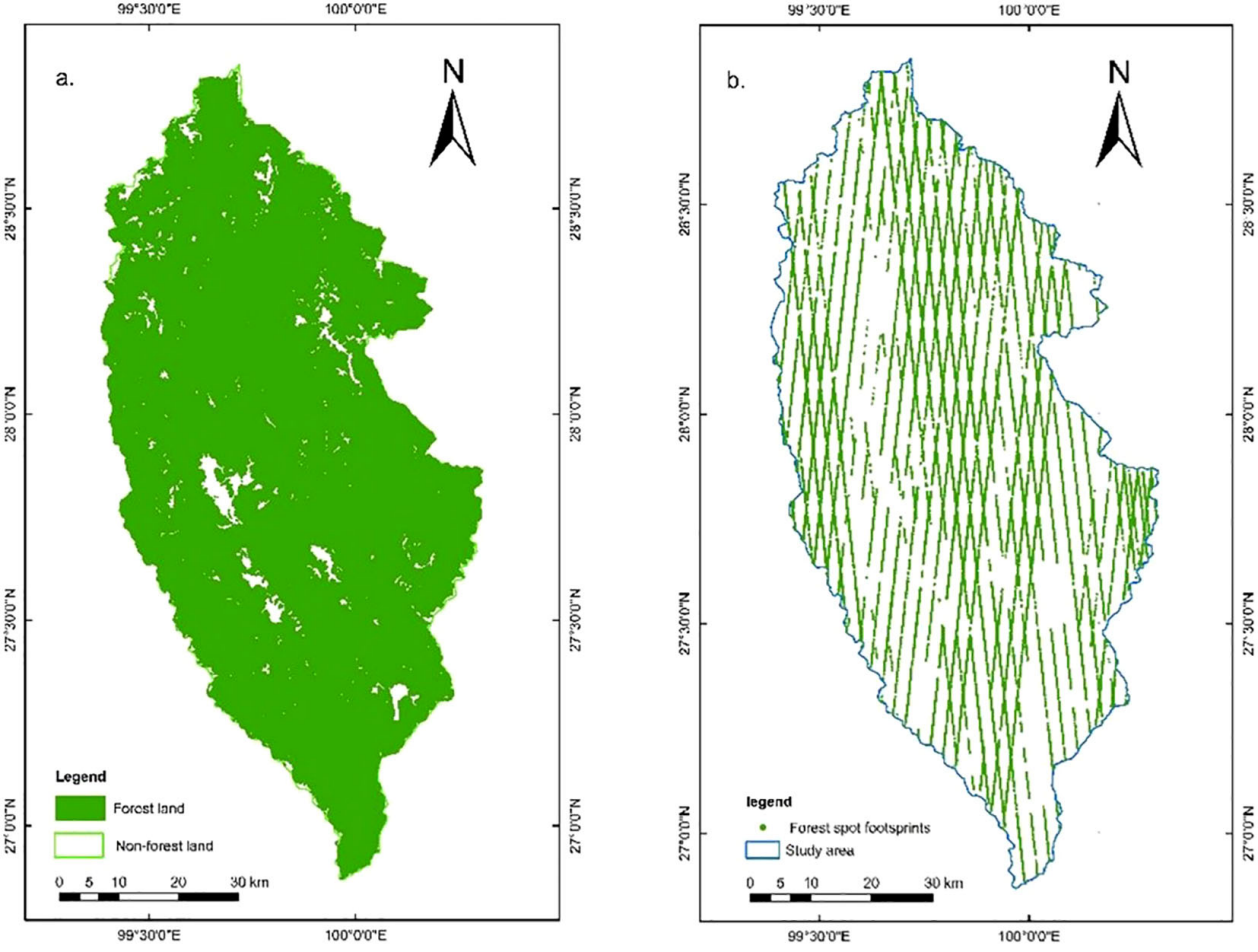
**(a)** Forest land distribution map. **(b)** Effective forest spot footprint distribution map.

After further photon point cloud denoising and classification, the number of effective photons of ATL03 reaches tens of millions. According to the ATL08 product, 94,039 effective footprints in the study area were obtained by thinning sampling in a 100-m section. The latest sub-compartment attribute data of forest resources survey in Shangri-La City (2016) were used for overlay analysis. In the study area, 74,808 effective forest footprints (**Figure 2**) and 19,231 non-forest footprints were obtained, a total of 50 parameters (including 54 standard footprints data) in the effective forest footprints were extracted, and the parameter introduction is detailed in the literature (Nie et al., 2018; Zhang et al., 2021a).

### 2.4 Preprocessing and feature variable extraction of regional-scale remote sensing data

#### 2.4.1 Preprocessing of regional-scale remote sensing data

**Figure 3:**
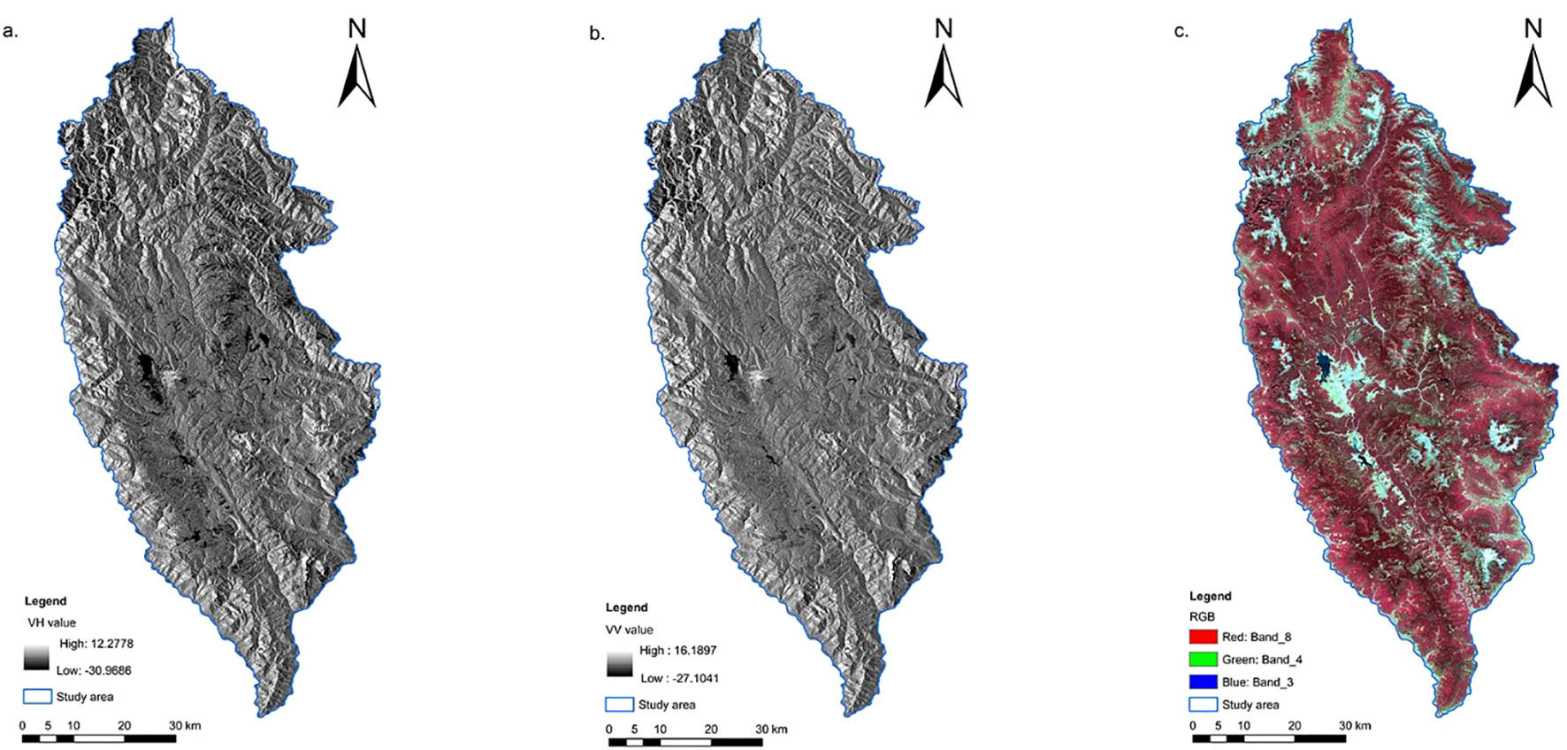
Backscatter images generated by Sentinel-1. **(a)** VH, **(b)** VV. **(c)** Standard false color image consisting of Band 8 (red), Band 4 (green), and Band 3 (blue) of Sentinel-2, with vegetation areas highlighted in red.

The study utilized Sentinel-1/2 images captured in October 2021, which were freely downloaded from the European Space Agency (ESA) (https://scihub.copernicus.eu/dhus/#/home) in November 2021. The SAR data include C-band dual-polarization (VV and VH) single-look complex data from the Sentinel-1A satellite, acquired in ground range detected (GRD) Level 1 product interferometric wide (IW) mode. The sensor operates at a center frequency of 5.405 GHz, with a swath width of 250 km and a spatial resolution of 15 m × 15 m after resampling. To extract the backscattering coefficient from dual-polarization backscatter images, SNAP (sentinel application platform) software was employed for data preprocessing steps including precise orbit determination, thermal noise removal, radiometric calibration, multi-looking, speckle filtering, geocoding, and dB conversion (**Figures 3a, b**). The Sentinel-2 L2A-level multispectral data used is a product of L1C-level images after Sen2cor atmospheric correction. Using SNAP software, each band is resampled to 15 m by three convolutions, and then 5-m SPOT-5 high-precision images are used for geometric correction, and the SCS + C model is used for topographic correction (**Figure 3c**).

#### 2.4.2 Regional-scale feature variables extraction

A total of 91 feature variables were extracted, including remote sensing factors such as texture features, vegetation indices, single-band reflectivity, SAR factors, and three terrain factors (**Table 2**). All feature variables were extracted using ENVI 5.6 software. VV/VH represents the ratio of VV to VH, while VV−VH denotes the difference between VV and VH. Texture features were generated using the gray-level co-occurrence matrix (GLCM) method within the second-order texture algorithm. The window size was set to 5 × 5, the step size was set to 1, and the gray level was set to 64, resulting in the extraction of eight texture features.

**Table 2:** Factor extraction of regional-scale feature variables.

Data sources	Variable type	Variable name	Number of variable
Sentinel-1	Backscattering coefficient	VV, VH, VV/VH, VV-VH	4
Sentinel-2	Single-band factor	Band 2, Band 3, Band 4, Band 5, Band 6, Band 7, Band 8, Band 8A	8
	Vegetation index	NDVI, DVI, SAVI, OSAVI, EVI, EVI2, RVI, MASVI, GNDVI, GRVI, RDVI, IDVI	12
	Texture feature	SE_ME, SE_VA, SE_HO, SE_CO, SE_DI, SE_EN, SE_SM, SE_CR	64
DEM	Topographic factors	Slope, aspect, elevation	3

SE_XXX represents the texture feature generated by the single band of Sentinel-2, SE represents the single band of B2-B8A, and XXX represents the texture feature.

### 2.5 Digital elevation model data

In this study, DEM data with a spatial resolution of 12.5 m were used to extract three topographic factors: slope, aspect, and elevation. The data were obtained from the polarimetric synthetic aperture radar (PolSAR) sensor aboard the ALOS satellite and were freely downloaded from the official earthdata website (https://www.earthdata.nasa.gov, accessed in November 2021).

## 3 Research methods

**Figure 4:**
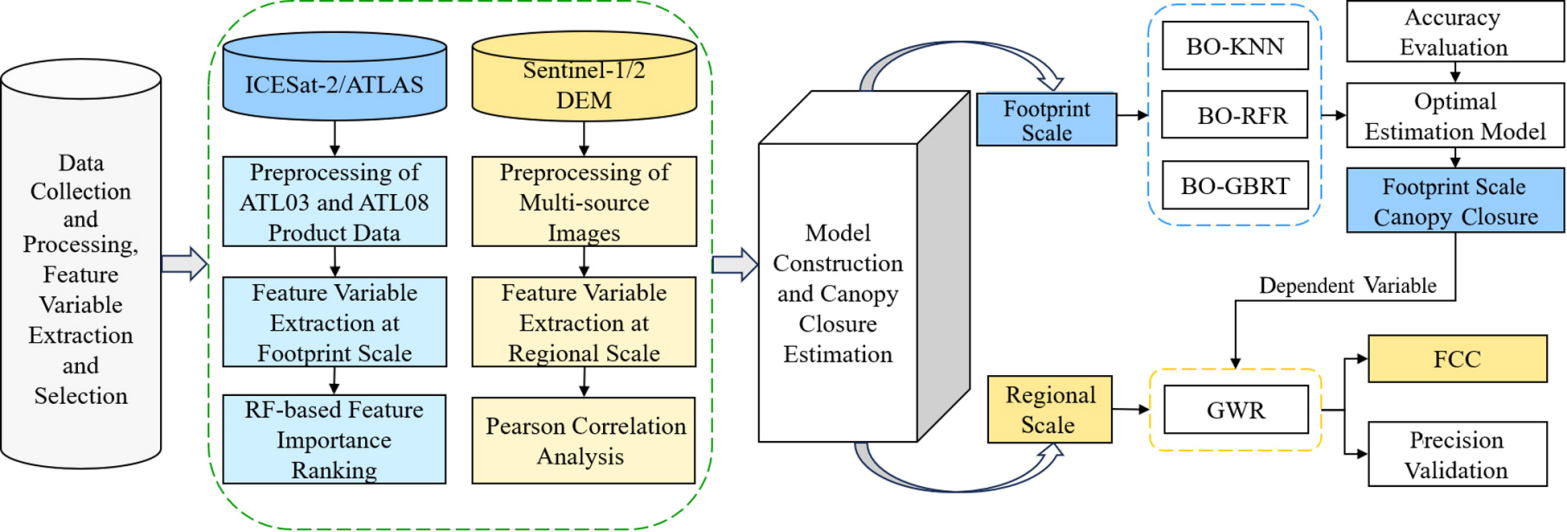
Technical route.

The methodology consists of four main steps (**Figure 4**): (1) dataset collection, preprocessing, and index extraction; (2) selection and modeling of footprint-scale characteristic variables and canopy closure estimation; (3) selection and modeling of regional-scale characteristic variables and FCC estimation for the study area; and (4) spatial mapping and analysis of FCC in the study area.

### 3.1 Bayesian optimization algorithm

The BO algorithm can obtain a global approximate optimal solution with little evaluation cost; in addition, the famous “Bayesian theorem” is used in the optimization process (Cui and Yang, 2018). The core is to use the probability model to represent the costly complex objective function of the original evaluation (Zhang et al., 2021b); the active selection strategy is constructed by using the posterior information of the surrogate model, that is, the acquisition function (Cui and Yang, 2018); this results in the probability model more accurately satisfying the behavior of the black box function and effectively reducing unnecessary sampling, thus theoretically ensuring the final convergence to the global optimal solution (Cui and Yang, 2018). In short, to reduce the model calculation amount and optimize the target model parameters, the model estimation accuracy should be improved. The Bayesian formula is as follows:


$$\begin{array}{c} \text{p}(\text{f}|{\text{D}}_{1:\text{t}})=\frac{\text{p}({\text{D}}_{1:\text{t}}|\text{f})\text{p}(\text{f})}{\text{p}({\text{D}}_{1:\text{t}})} \end{array}$$

where *f* represents the unknown objective function (parameters in the optimization model), $D_{1:t}=\{(x_{1},y_{1}),(x_{2},y_{2}),\ldots ,(x_{t},y_{t})\}$ represents the observed set, $x_{t}\;$ represents the decision vector, $y_{1}=f(x_{t})+\epsilon_{t}$ represents the observed value, $\epsilon_{t}$ represents the observation error; $p(D_{1:t}|f)$ represents the likelihood distribution of *y*, due to the error of the observed value, p(f) represents the prior probability distribution of *f*, that is, the assumption of the unknown objective function state, $p(D_{1:t})$ denotes the marginal likelihood distribution of the marginalized *f*, which is mainly used in BO for hyperparameters, $p(f|D_{1:t})$ represents the posterior probability distribution of *f*, and the confidence of the unknown objective function after the prior is corrected by the observed dataset.

**Figure 5:**
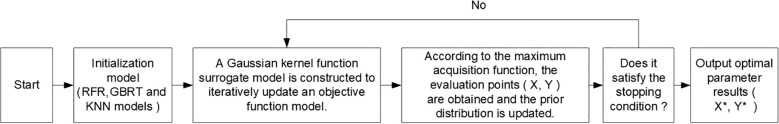
BO-RFR, BO-GBRT, and BO-KNN algorithm flowchart.

The BO process is an iterative process (Cui and Yang, 2018), and the optimization framework is shown in **Table 3**. There are three core steps: (1) Select the next evaluation points with the highest “potential” $\;x_{t}$ according to the maximum acquisition function. (2) Calculate the objective function value $y_{\text{t}}=f(x_{t})+\epsilon_{t}\;$ according to the selected evaluation point $\;x_{t}$ . (3) The newly obtained input–observation pair $\left\{x_{t},y_{t}\right\}$ is added to the historical observation set $D_{1:t-1}$ , and the probabilistic surrogate model is continuously updated to prepare for the next model iteration. The research mainly optimized the important parameters of random forest regression (RFR), gradient boosting regression tree (GBRT), and K-nearest neighbor (K-NN) models for 1,000 times to find the best parameters for modeling. The algorithm flow is shown in **Figure 5**.

**Table 3:** Framework of the bayesian optimization algorithm.

Algorithm: Bayesian optimization algorithm
Input: proxy model *f*, acquisition function *α*
Output: hyperparameter result X*
1: Initialize the hyperparameter x
2: for *t* = *1, 2…., T* do
3: Maximize the acquisition function to get the next evaluation point: $x_{t}=argmax_{x\in X}\alpha (x|D_{1:t-1})$
4: Evaluate the objective function value $\;y_{1}=f(x_{t})+\epsilon_{t}$
5: Integrate data: $D_{t}=D_{t-1}\cup \{x_{t},y_{t}\}$ , and update the proxy model
6: end for

### 3.2 Footprint-scale estimation model

In this study, RFR, GBRT, and K-NN models were selected as the basic models for footprint-scale FCC estimation. The introduction of each model is shown in various studies (Franco-Lopez et al., 2001; Coulston et al., 2016; Tian et al., 2021). The BO algorithm was used to optimize the three basic models to find the best kernel parameters for modeling and accurately estimate the footprint-scale FCC. The optimization parameters are shown in **Table 4**.

**Table 4:** Description of RF, GBRT, and K-NN model parameters.

Model type	Parameters	Description	Type
RF, GBRT	max_depth	The maximum depth of the tree	int
	n_estimators	The number of trees in the forest	int
	min_samples_split	The minimum number of samples required to split an internal node	int or float
	min_samples_leaf	The minimum number of samples required to be at a leaf node	int or float
K-NN	n_neighbors	The number of neighbors to use by default for k-neighbors queries	int
	weights	The weight function used in prediction	Str or callable

### 3.3 Regional-scale estimation model of geographically weighted regression model

In GWR , the locally weighted least squares method is used to solve for local parameters (Guo et al., 2012; Zhang et al., 2019b), with weights calculated based on the spatial distance between the location to be estimated and the locations of other observation points (Nazeer and Bilal, 2018). As an extension of the linear regression model, GWR has strong spatial variability and correlation in spatial position, which can effectively explain the influence of different independent variables on target variables in different spatial positions. The mathematical model of GWR is as follows (Song et al., 2022c):


$$\;\begin{array}{c} {\text{y}}_{\text{i}}={\text{a}}_{0}({\text{u}}_{\text{i}},{\text{v}}_{\text{i}})+\sum_{\text{k}}{\text{a}}_{\text{k}}({\text{u}}_{\text{i}},{\text{v}}_{\text{i}}){\text{x}}_{\text{i}k}+{\text{ϵ}}_{\text{i}} \end{array}$$

where $y_{i}$ is the target variable of point *i*, $x_{ik}$ is the value of the *k* independent variable in *i*, *k* is the independent variable count, and *i* is the sample point count, $\epsilon_{i}$ is the residual error, $\left(u_{i},v_{i}\right)$ is the spatial coordinates of the *i* sample point, $a_{k}(u_{i},v_{i})$ is the local regression coefficient at point *i*, that is, the spatial location function.

Because the research data are continuous, the Gaussian kernel function model is selected to construct the spatial weight matrix, and the calculation formula is as follows (Nazeer and Bilal, 2018):


$$\begin{array}{c} {\text{w}}_{\text{i}j}=\text{exp}(-\frac{d_{\text{i},\text{j}}^{2}}{{\text{θ}}^{2}}) \end{array}$$

where i,j denotes the spatial location of the regression point, $w_{ij}\;$ is the weight value of the observation at the position *j* representing the coefficient at point *i*, $d_{i,j}$ is the Euclidean distance between *i* and *j*, θ is the fixed bandwidth size defined by the distance metric.

As GWR is weak in the diagnosis of independent variable factors; OLS is needed for the collinearity diagnosis and significance test of independent variable factors to judge the feasibility of constructing GWR, to select the independent variable factors that fit the GWR model, and finally to improve the accuracy of the GWR model and to construct a more realistic regression model.

### 3.4 Evaluation of model accuracy

In this study, the coefficient of determination (*R* ^2^), root mean square error (RMSE), mean absolute residual (MAR), and prediction accuracy (*P*) of the leave-one-out cross validation (LOOCV) method were used to verify the prediction accuracy of the estimation model (Shu et al., 2022; Song et al., 2022b). This method is applied to small sample data for sequential training and verification, addressing local optimization issues and enhancing model robustness (Shu et al., 2022). Additionally, compared to K-fold and holdout cross-validation, LOOCV is not influenced by random factors (Song et al., 2022b), thereby reducing the uncertainty of model estimation results. The calculation formula is as follows:


$$\begin{array}{c} {\text{R}}^{2}=\frac{\sum_{\text{i}}^{N}{\left({\hat{\text{y}}}_{\text{i}}-\bar{\text{y}}\right)}^{2}}{\sum_{\text{i}}^{N}{\left({\text{y}}_{\text{i}}-\bar{\text{y}}\right)}^{2}} \end{array}$$

$$\text{RMSE}=\sqrt{\frac{\sum_{\text{i}}^{N}{\left({\text{y}}_{\text{i}}-{\hat{\text{y}}}_{\text{i}}\right)}^{2}}{\text{N}}}$$

$$\begin{array}{c} \text{P}=(1-\frac{\text{RMSE}}{\bar{\text{y}}})\times 100\% \end{array}$$

$$\begin{array}{c} \text{MAR}=\frac{1}{\text{n}}\sum_{\text{i}=1}^{\text{n}}|({\text{y}}_{\text{i}}-{\hat{\text{y}}}_{\text{i}})| \end{array}$$

where ${\hat{y}}_{i}$ is the model prediction value; $\bar{\text{y }}\;$ is the average model prediction value; ${\text{y}}_{\text{i}}\;\;$ is the canopy closure measured value; and *N* is the total number of verification samples.

## 4 Results and analysis

### 4.1 Optimization results of characteristic variables

#### 4.1.1 Optimization results of footprint-scale characteristic variables

In this study, the extracted 50 parameters values were used to evaluate the importance of features using RF (Glenn et al., 2016; Chen et al., 2022). All parameters have a certain contribution rate. The 5% of the importance of features was set as a threshold, and six feature variables were selected as the best independent variables for modeling (**Table 5**). Among them, Landsat_perc has the highest feature contribution of 12.68%, and asr has the lowest feature importance of 5.03%.

**Table 5:** Statistics of the results of ICEsat-2/ATLAS feature variable preferences.

Variable name	Description	Value (%)
landsat_perc	Average percentage value of the valid Landsat Tree Cover Continuous Fields product for each 100-m segment	12.68%
toc_roughness	Standard deviation of the relative heights of all photons classified as top of canopy within the segment	6.47%
h_min_canopy	The minimum of relative individual canopy heights within segment. Relative canopy heights have been computed by differencing the canopy photon height from the estimated terrain surface	6.06%
h_dif_canopy	Difference between h_canopy and h_median_canopy	5.38%
n_toc_photons	The number of photons classified as top of canopy within the segment	5.37%
asr	Apparent surface reflectance	5.03%

#### 4.1.2 Optimization results of regional-scale characteristic variables

The remaining 13 independent variables after the preferential selection of the 91 regional-scale characteristic variables extracted using Pearson correlation analysis (Duncanson et al., 2020) had correlations greater than 0.2 and significant at the 0.01 level (**Table 6**). Among them, the average correlation of the GLCM generated by the green edge band and the red edge band was strong, which may be related to the fact that the texture feature factors can describe more detailed forest structure information (Shu et al., 2022). This result is consistent with the results of  Zhang et al. (2016) in the Daxing’anling area of Inner Mongolia. The maximum correlation coefficients of B3_DI and B3_HO were 0.338 and 0.338, respectively, and the minimum correlation coefficient of GNDVI was 0.22. The VV−VH correlation coefficient based on the difference between the backscattering coefficient VV and VH in the SAR factor is −0.287. Among the terrain factors, only the slope meets the preferred standard, and the correlation coefficient was 0.336.

**Table 6:** Multi-source remote sensing factor preference results statistics.

Variable name	Correlation coefficient	Variable name	Correlation coefficient	Variable name	Correlation coefficient
Slope	0.336**	B3_HO	−0.338**	B7	0.251**
VV-VH	−0.287**	B8_SM	−0.327**	NDVI	0.286**
B3_SM	−0.329**	B8_EN	0.293**	GNDVI	0.220**
B3_EN	0.307**	B6_ME	0.287**		
B3_DI	0.338**	B8A_CR	0.271**		

** is expressed significant at the 0.01 level.

### 4.2 ICESat-2/ATLAS footprint-scale indefinite FCC modeling results

**Figure 6:**
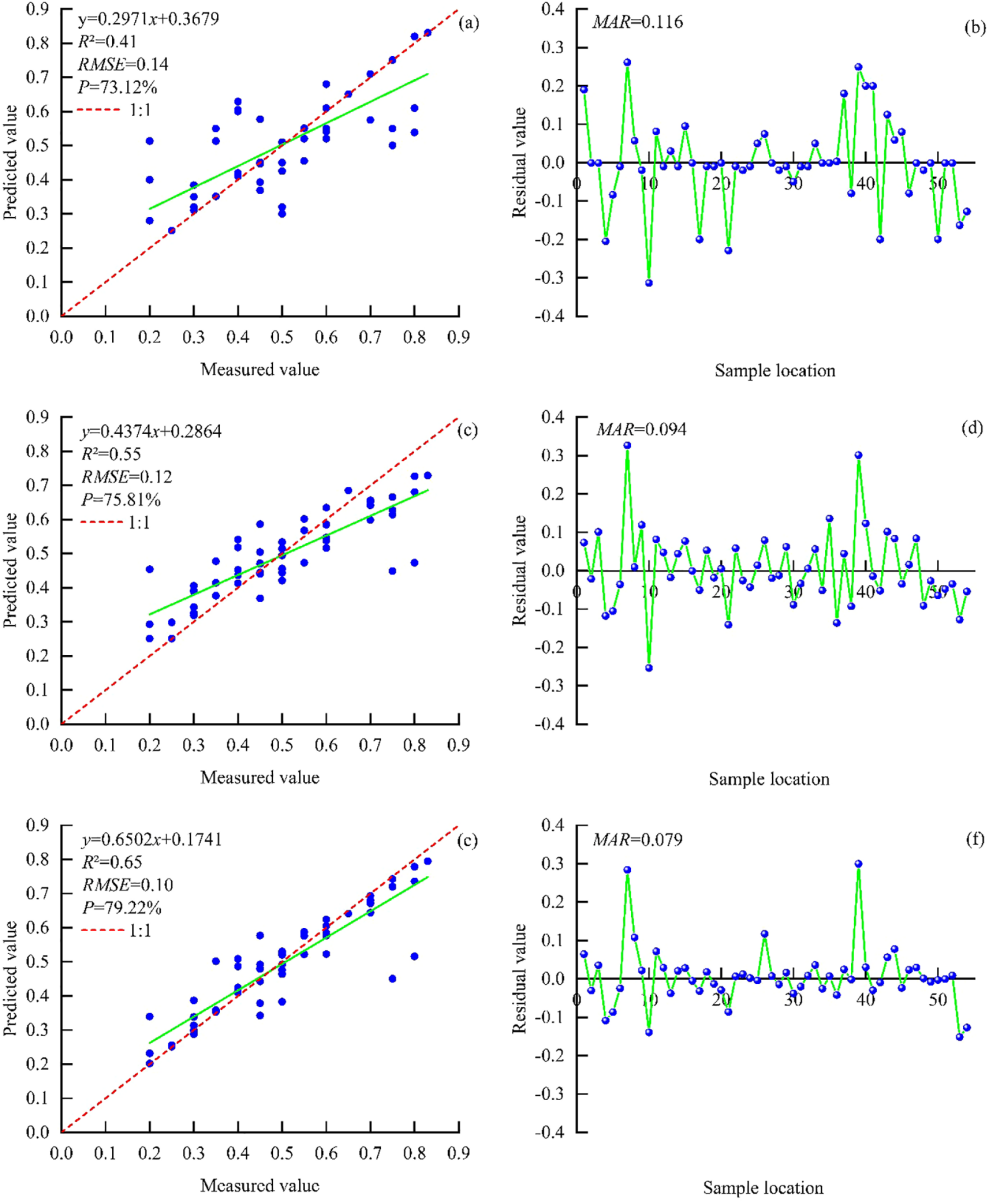
FCC shows the accuracy and residual footprint scale of the estimate model with the BO-KNN **(a, b)**, BO-RFR **(c, d)**, and BO-GBRT **(e, f)** models.

Six independent variables selected by ATLAS parameters were used to participate in the modeling to construct the best FCC estimation model of footprint scale. According to the model accuracy (**Figures 6a, c, e**), the overlap of multiple values in the same interval is caused by the fact that there are multiple measured values that are equal in different places in 54 sample plots. In the modeling results, there is a significant change in model accuracy for the nonparametric model before and after optimization using the BO algorithm. Before optimization (**Table 7**), the K-NN, RFR, and GBRT models had *R* ^2^ between 0.23 and 0.30, an RMSE range of 0.14–0.16, and a *P* range from 67.26% to 72.73%. After optimization (**Table 7**), the BO-KNN, BO-RF, and BO-GBRT models had *R* ^2^ between 0.41 and 0.65, with an average increase of 48.95% compared with before, an RMSE range of 0.10 to 0.14, with an average error reduction of 20.36% over the previous ones, and a *P* of 73.12% to 79.22% with an improved accuracy of 5.93% compared to the previous average. Among them, the BO-RFR and BO-GBRT models had the best fitting degree. The BO-GBRT model had the highest *R* ^2^ (0.65), a minimum RMSE (0.10), and the highest *P* (79.22%); hence, the comprehensive evaluation of the model was better. The optimized model residual diagram reflected the deviation between the measured value and the predicted value, and the fluctuation range of residual (**Figures 6b, d, f**) was between −0.3 and 0.4. **Figure 6b** fluctuated greatly, and the mean absolute residual reached 0.116. The change trend of **Figures 6d, f** was similar, and the minimum MAR of **Figure 6f** was 0.079; moreover, the error between the measured value and the predicted value was smaller. In summary, the BO-GBRT model had the best comprehensive fitting accuracy, which is selected as the best estimation model for footprint-scale FCC.

**Table 7:** Modeling results of the footprint-scale depression estimation model.

	Model type	*R* ^2^	RMSE	*P* (%)
	KNN	0.23	0.16	67.26
Un-optimized	RFR	0.28	0.15	70.31
	GBRT	0.30	0.14	72.73
	BO-KNN	0.41	0.14	73.12
Optimized	BO-RFR	0.55	0.12	75.81
	BO-GBRT	0.65	0.10	79.22

### 4.3 The spatial distribution of FCC in footprint scale

**Figure 7:**
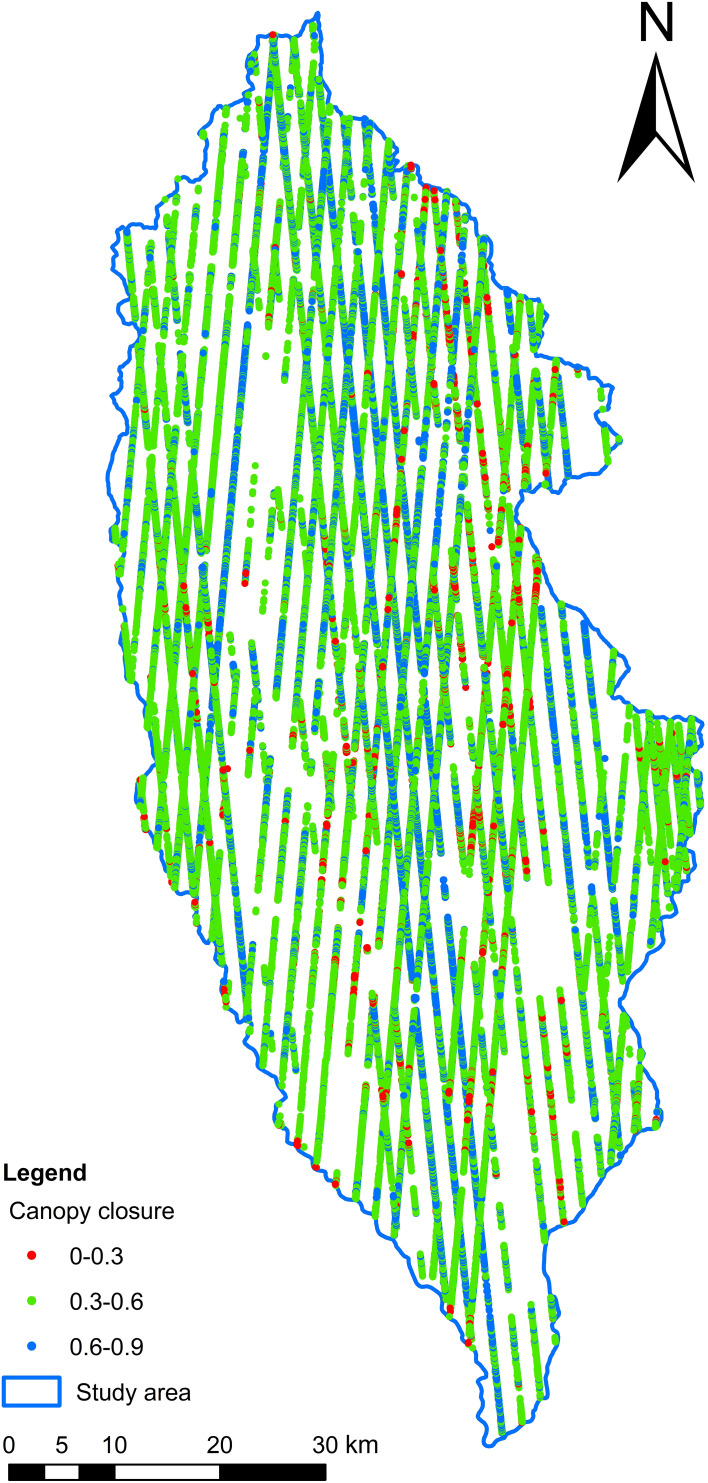
Spatial distribution of footprint FCC in the study area.

The spatial distribution of FCC values within the ATLAS footprints in the study area was estimated using the BO-GBRT model (**Figure 7**). FCC was mainly concentrated in the range 0.3–0.6, a few were distributed in the range 0.6–0.9, and a very small part was between 0 and 0.3. The overall spatial distribution of the depression FCC value in the footprint varied greatly, and the distribution in local areas was relatively uniform. The high-value footprints of depression FCC in the study area were distributed from southeast to north, and the northern area was the main distribution area of FCC high value with relatively uniform distribution while the southern and south-central areas had more distribution of FCC low-value footprint. As the terrain of the study area is high in the northwest and low in the southeast, the climatic conditions in the southeast are more suitable than those in the northwest and north, where human settlements are found, with a lower FCC value (Song et al., 2022b; Yu et al., 2024). This illustrated the feasibility of using ICESat-2/ATLAS data for estimating FCC.

### 4.4 The spatial distribution of FCC in the study area

#### 4.4.1 Diagnosis of independent variable factors based on OLS and normal test results

In order to eliminate the influence of multicollinearity among multivariate factors on the GWR model, the 13 preferred multi-source remote sensing factors were analyzed for covariance diagnosis using OLS. The independent variables with variance inflation factor (VIF) greater than 10 were deleted, and the remaining six independent variable factors (**Table 8**) were significant at the 0.01 level. Among them, when the VIF value of vegetation index was within the range of 6–7, the VIF values of slope factor, SAR factor, and texture feature factor were close to 1.

**Table 8:** OLS covariance diagnosis table for the independent variable factors.

Variable name	NDVI	GNDVI	B8_SM	B8A_CR	VV-VH	Slope
VIF	6.70	6.88	1.01	1.09	1.05	1.09

**Figure 8:**
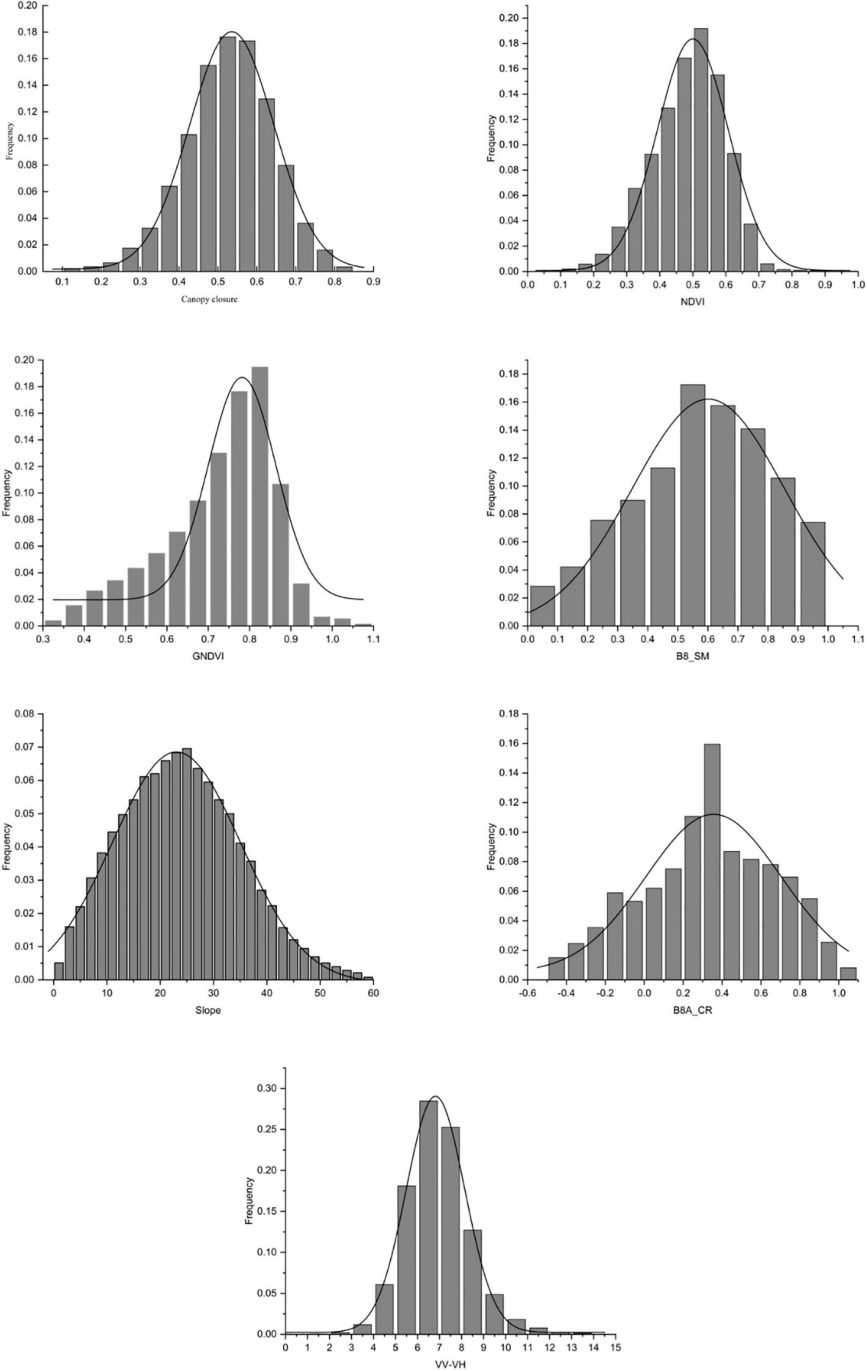
Frequency distribution histogram of each variable factor. Canopy closure, NDVI, GNDVI, B8_SM, Slope, B8A_CR, and VV-VH.

The premise of using the GWR model to construct a mathematical model is that the experimental data must conform to normal distribution (Guo et al., 2012; Zhang et al., 2019b). The frequency distribution histogram of the FCC and the six independent variable factors were tested by the data, showing a bell-shaped curve (**Figure 8**), which conformed to the normal distribution.

#### 4.4.2 Prediction results and verification of the GWR model

**Figure 9:**
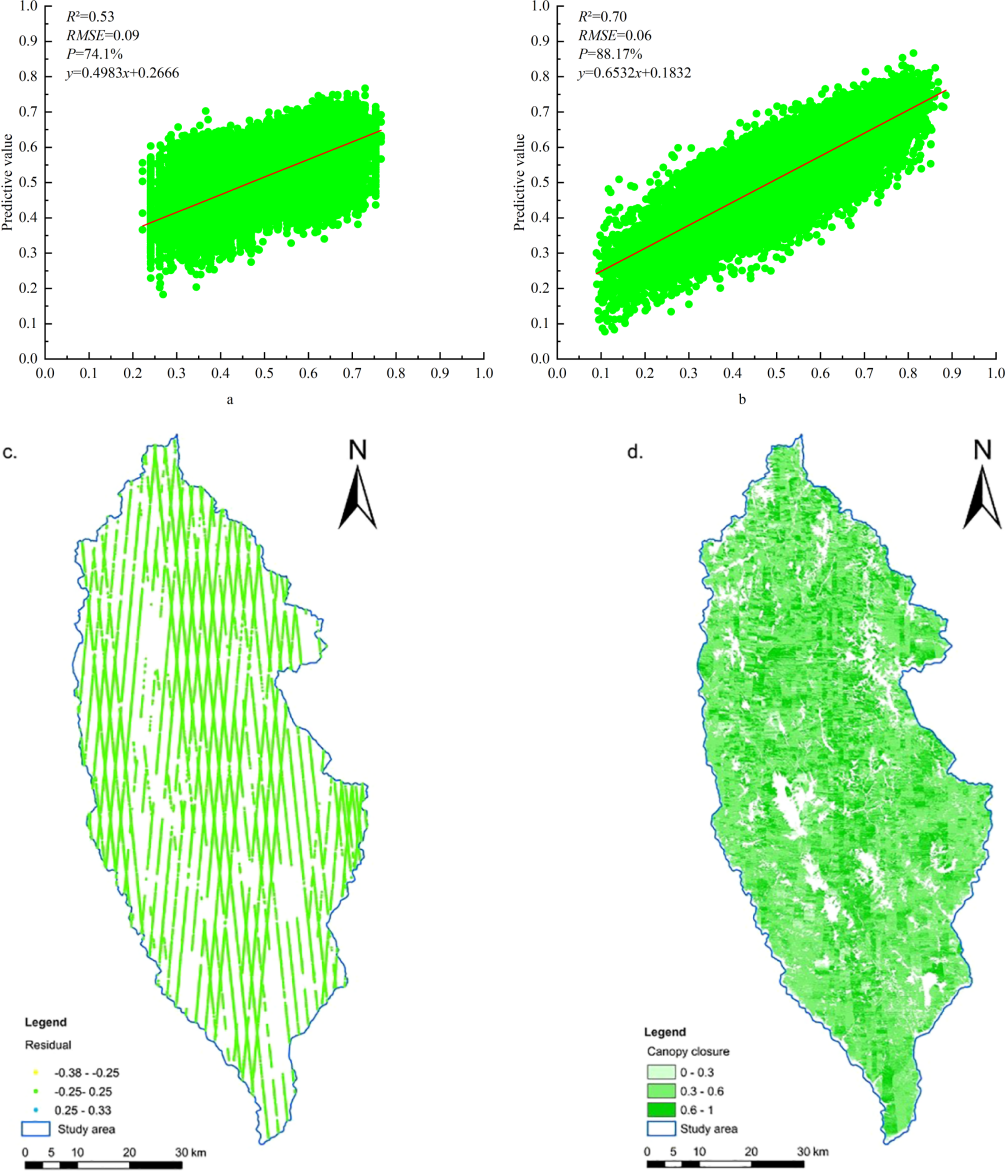
Accuracy of GWR model fit and estimation results. **(a)** Before optimization. **(b)** After optimization. **(c)** Residual distribution. **(d)** Spatial distribution of FCC in the study area.

NDVI, GNDVI, B8_SM, B8A_CR, VV-VH, and Slope were used as dependent variables; meanwhile, the GWR model tool provided by ArcGIS Pro was used to predict FCC at the regional scale. The bandwidth type is the number of neighbors, and the neighborhood method selects the golden search to find the minimum corrected Akaike information criterion (AICc) to determine the optimal bandwidth. Further AICc represents a measure of the model, and a smaller value indicates that the fitted mathematical model is better (Guo et al., 2012), and the local weight is double squared. According to **Figure 9a**, the FCC value of ATLAS footprints estimated by the GBRT model was used as the dependent variable of the GWR model, with a GWR model validation accuracy of *R* ^2^ = 0.53, RMSE = 0.09, and AICc = 106,917.29. While using the ATLAS footprints, the FCC value is estimated by the BO-GBRT model as the dependent variable of the GWR model (**Figure 9b**), and the GWR model validation accuracy is *R* ^2^ = 0.70, which is 32.08% better than before optimization. RMSE = 0.06 is reduced by 33.33% compared to that of the comparison before optimization, and the AICc = 99,265.12, which is 7.16% lower than that before optimization. The optimized model residuals were mainly distributed in the range of −0.25 to 0.25 (**Figure 9c**), the *MAR* was 0.047, and the local *R* ^2^ was mainly distributed in the range of 0.3–0.6 with an average of 0.50, and the estimation model has high accuracy. At the same time, while handling the large number of modeling samples, it is feasible to use the GWR mathematical model fitted with six explanatory variables to predict the FCC value of the unknown spatial region (**Figure 9d**).

According to **Figure 9d**, the average value of FCC was 0.50, and the values were mainly distributed between 0.3 and 0.6, accounting for 68.43%, followed by 0.6–1, accounting for 22.48%, and by 0–0.3, accounting for 9.09%. The areas with low estimates of depression FCC in the study area were mainly distributed at the margins, mostly in the river or perennial snow-covered areas, and in the southeastern urban areas where humans gathered (Song et al., 2022a). The area with high depression FCC runs through from northwest to southeast, and the northern area was the main distribution area with high depression FCC, mainly due to the increase in plantation forest area in the central and northern regions, while the northeast region was the distribution area of the Pudatso National Forest Park (Shi et al., 2015; Su et al., 2020), which confirmed the reliability of the results of forest depression in the study area estimated by the GWR model. Moreover, Shangri-La is an alpine mountain area in the study area, which belongs to the national ecologically fragile area, and the logging of natural forests has been prohibited since 1998, and there has been no relevant management and logging activities in the past 20 years; hence, the results had certain credibility.

**Figure 10:**
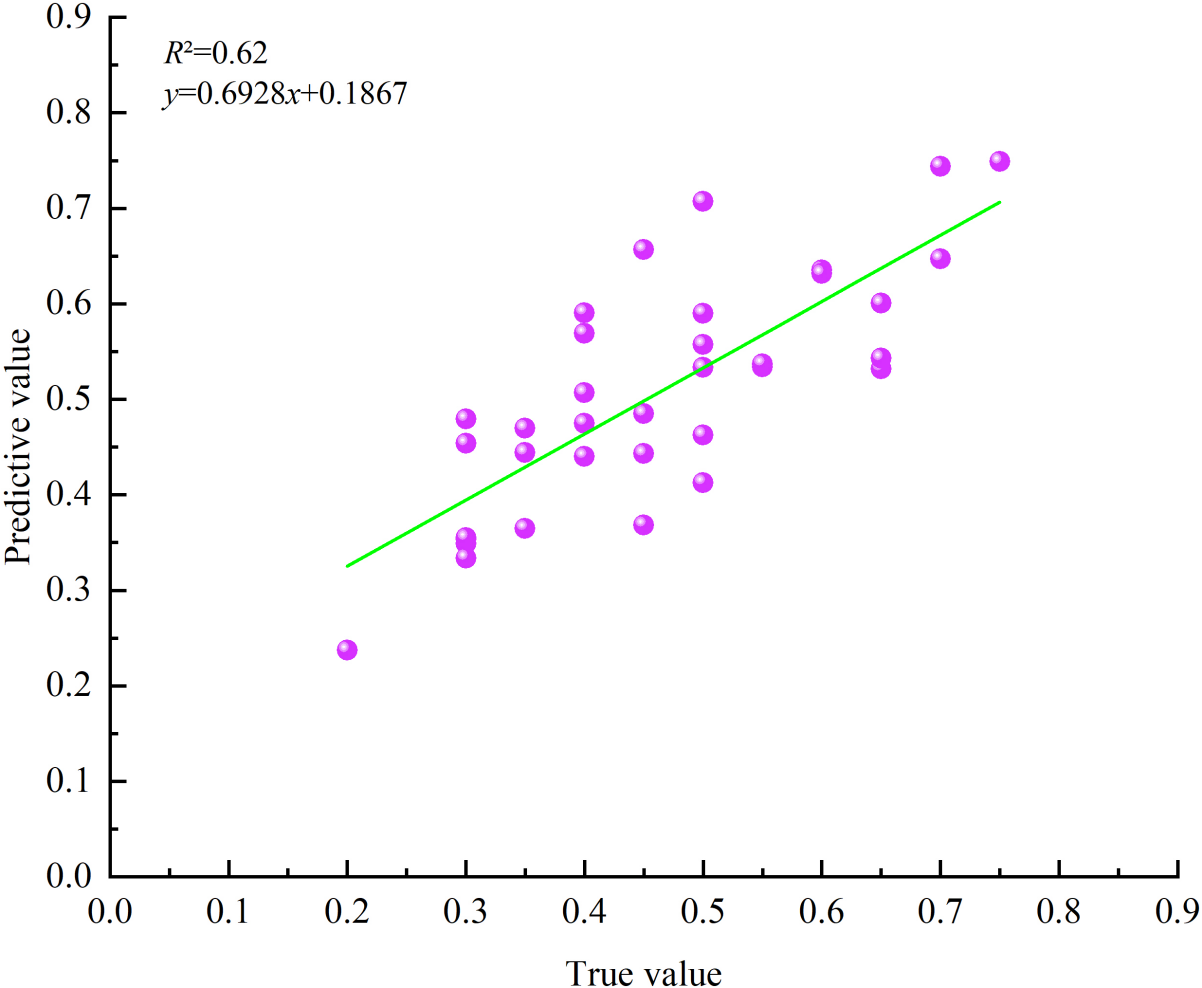
Linear model fitting diagram of measured value and predicted value.

The prediction results of the GWR model were verified using data from 40 sample plots (20 × 20 m) from the field survey in the study area in November 2016 (**Figure 10**). It is verified that the *R* ^2^ between the FCC predicted value and the measured value is 0.62, and the Pearson correlation coefficient is 0.784 (significant at the 0.01 level), which has high consistency. It indicated that the method of estimating FCC in the study area by using the ATLAS footprint FCC value as the training sample data of the GWR model and cooperating with multi-source remote sensing factors was feasible, and the estimation results were reliable.

## 5 Discussion

### 5.1 Model error propagation and Bayesian optimization algorithm

Fu et al. (2014) and Qin et al. (2017) showed the uncertainty of aboveground biomass and carbon storage in forests, respectively. The uncertainty of the remote sensing model was the main error source that causes the uncertainty of biomass and carbon storage estimation, and the accuracy of the model plays an important role in the estimation results. In this study, the FCC value estimated by the footprint scale is used as the training sample of the regional-scale GWR model. In the scale-up process, there is model error transmission. In order to weaken the influence of this error on the regional-scale FCC estimation results, the BOA is used to further optimize the three initial machine learning models, so as to optimize the optimal FCC estimation model of footprint scale and improve the estimation accuracy of the model. This study shows that after using BO to optimize the initial machine learning model, the accuracy of the model has been significantly improved compared with that before optimization. However, the FCC value of ATLAS footprints estimated by the BO-GBRT model was used as the dependent variable of the GWR model. Compared with before optimization, *R* ^2^ increased by 32.08%, RMSE decreased by 33.33%, AICc decreased by 7.16%, and *P* increased by 14.07%. Therefore, BOA can effectively improve the accuracy of the estimation model and weaken the influence of model error transfer on the FCC estimation results. In this study, the model fitting accuracy is BO-GBRT > BO-RFR > BO-KNN. The reason is that the K-NN model was suitable for large samples because of non-assumptions on the data and non-sensitivity to abnormal samples (Shu et al., 2022), and the GBRT model was based on the iterative improvement of the original model, so that the next new model had a smaller error than the previous model, and a new combined model was established in the gradient direction of the residual reduction, which often has higher fitting accuracy than RF (Zhang et al., 2019a; Yu et al., 2021). However, the BOA was only used to optimize the main parameters of the original model for 1,000 times. In order to further improve the estimation accuracy of the model, the comprehensive search algorithm can be introduced to optimize all the parameters of the model (Pedregosa et al., 2011), or introduce algorithms such as deep forest so that small sample data can also be fitted by neural network learning (Xia et al., 2022).

In the extrapolation model of FCC in the study area, the AICc value of the GWR model is too large before and after optimization as the sample size was too large, and the growth of the maximum likelihood estimation of the variance of the random error term may slow down (Zhang et al., 2019b). The spatial distribution of the weight of the independent variable with the FCC will change due to the geographical location, showing the local spatial dependence and heterogeneity of the independent variable index; therefore, the GWR model can well combine the spatiality between the independent variable indicators to predict the canopy closure of the unknown space (Guo et al., 2012), specifically the canopy closure *P* that reached 88.27%, and the RMSE was 0.06.

### 5.2 Characteristic variable setting and selection

The optimization and combination of characteristic variable factors determine the accuracy of the prediction model and inversion results to a certain extent (Zhang et al., 2022). In the study, the more advanced photon-counting LiDAR data were used in the feature variable setting of the footprint scale (Lin et al., 2020; Zhu et al., 2020), and the spectral saturation point is higher. RF was used to select six characteristic variables with the largest contribution to construct the footprint-scale FCC estimation model. In the selection of regional-scale feature variables, optical remote sensing data are affected by the “light saturation” characteristics of forest vegetation to varying degrees. Among them, the single-band reflectance had the greatest impact, followed by the vegetation index, while the texture feature can represent ground object structure information in remote sensing images, reflecting the important information of spatial changes of land cover type in remote sensing images (Shu et al., 2022) and the forest structure information, which is least affected by “light saturation” (Zhao et al., 2016). In this paper, the integration of multi-sensor and auxiliary data is realized by adding SAR factors and terrain factors to solve the problem of data saturation (Zhao et al., 2016). At the same time, considering the collinearity problem among the independent variables of the GWR model (Guo et al., 2012; Nazeer and Bilal, 2018; Zhang et al., 2019b; Song et al., 2022a), the same type of factors should not be too much, so the study only selected the sliding window of 5×5 texture feature factors. Finally, after correlation analysis, OLS test, and normal transformation, six explanatory variables were selected to construct the GWR mathematical model, which reduced the saturation problem of remote sensing data to a certain extent and improved the model estimation accuracy.

### 5.3 Transplantability of FCC estimation models in different study areas or forest types

In this paper, a process-oriented programming processing module was established for ATLAS data processing and parameter batch extraction, feature variable preference, optimization of the main parameters of the model, and optimization of multiple nonparametric models to fit the best model. Xie et al. (2022) used multispectral satellite images in Google Earth Engine to improve the estimation of FCC. The results showed that the calibration of model parameters needed to determine the range of values manually. The advantage of this study is that researchers only need to input ATLAS data and measured sample data for modeling, and select the best model according to their own needs to estimate all the FCC prediction values in the regional footprints. Based on the characteristics of ICESat-2/ATLAS data (Neuenschwander and Pitts, 2019), full domain coverage of global regions can be basically achieved to meet the needs for flexible selection of different study areas and model portability testing. Zhu et al. (2020) analyzed the forest height based on different modes of spaceborne LiDAR data, and the results showed that the forest height consistency model established in different forest types or experimental area data was universal, and the model accuracy was consistent with the original model accuracy. The dataset used in the study was affected by complex terrain and high-altitude factors. However, the vegetation community in the study area was not distinguished and only relative sampling was carried out, and the sample size was 54, which had met the principle of large samples in field investigation (Shu et al., 2022). In future studies, the sample size of different slopes and different community compositions can be increased to explore their effects on the estimation results separately. At the same time, the same experimental design of different communities can be set up in low-altitude and relatively flat areas such as plains and hills to verify the portability of the model and obtain more accurate FCC estimation results. This provided not only a reliable reference for the study of energy transfer and microclimate changes in global forest ecosystems and forest tending evaluation but also a way of thinking for characterizing FCC maps on a global scale.

### 5.4 The influence of mountain terrain on FCC estimation results

The complex terrain area had a certain impact on the FCC estimation results: the larger the terrain slope, the greater the influence of the vegetation canopy on the laser echo. Compared with ICESat-1/GLAS data (Wang et al., 2015; Cui et al., 2021), the new-generation ICE-Sat-2/ATLAS data were used in this study; the footprint diameter is only 17 m and the footprint interval is 0.7 m, which greatly reduced the influence of terrain on footprint echo (Moudrý et al., 2022) and improved the accuracy of model estimation. The data of 54 measured plots used in this study showed that the proportion of plots with a slope of 0°–10°, 10°–20°, and greater than 20° was 42.59%, 29.63%, and 27.78%, respectively, and the slope distribution was relatively uniform. Based on this, a forest ATLAS footprint FCC estimation model was constructed, in which the model had high verification accuracy (*R* ^2^ = 0.65, RMSE = 0.10, *P* = 79.2%, MAR = 0.079) and can be used as a mountain FCC estimation model. This provides the possibility for footprint-scale low-cost ground plot survey to achieve accurate regional-scale FCC estimation.

## 6 Conclusions

In order to evaluate the ability of spaceborne photon-counting radar to estimate FCC, ICESat-2/ATLAS was used to obtain photon point cloud data. After denoising and classification of the photon point cloud, the parameter values (including 54 measured sample data) were extracted; meanwhile, six feature variables were selected by RF, and the best footprint-scale FCC estimation model was constructed based on BO-RFR, BO-KNN, and BO-GBRT to obtain the FCC value of ATLAS footprints. Additionally, the training sample data of the GWR model and the continuous planar FCC products in the whole study area are predicted. The main conclusions are as follows:

1. After the pretreatment of ICESat-2/ATLAS data, the extracted parameters had an ideal way of estimating FCC. Among them, the BO-GBRT model had the best verification accuracy as the best footprint scale FCC estimation model (*R* ^2^ = 0.65, RMSE = 0.10, *P* = 79.22%, MAR = 0.079). It showed that the BO algorithm can improve the model fitting accuracy, and the best estimation model compared with a variety of nonparametric models can reduce the influence of model error transfer on the FCC estimation results. The parameter with the largest contribution rate to the model was landsat_perc at 12.68%.
2. The ATLAS footprint FCC was used as the training sample of the regional-scale GWR model, and the FCC results were better in the study area with Sentinel 1/2 images and topographic factors. A total of 91 feature variables were extracted from DEM and multi-source remote sensing images for feature optimization, and 13 independent variables were retained. After OLS test and normal transformation, a total of six explanatory variables were involved in the mathematical model construction of GWR, and the final model verification accuracy was *R* ^2^ = 0.70, RMSE = 0.06, and *P* = 88.27%. The VV–VH factor was calculated based on the difference between VV and VH, which had good adaptability to the model, and the correlation was −0.287. The texture feature factors B3_DI and B3_HO had the strongest correlation with FCC.
3. The results of the study area estimated by the GWR model were used for spatial mapping, and the FCC distribution in the study area was consistent with the distribution of footprint-scale FCC. The *R* ^2^ between measured value and predicted value of the sample was 0.65, and the correlation coefficient was 0.784, which had high consistency. The average value of FCC was 0.50, and the values were mainly distributed between 0.3 and 0.6, accounting for 68.43%, followed by 0.6 to 1, accounting for 22.48%, and by 0 to 0.3, accounting for 9.09%. The high value area of FCC was distributed from northwest to southeast. The northern and southeastern regions were the main distribution areas of high and low FCC values, respectively, which is highly consistent with the forest distribution in the study area. The research showed that it is feasible to use ICESat-2/ATLAS data to predict the FCC value of ATLAS footprints based on the optimized machine learning method and to use such data as the training sample data of the GWR model, combined with multi-source remote sensing factors to estimate regional FCC. In summary, this provides a new scientific method for obtaining large-scale FCC with low cost and high precision.

## Data Availability

All satellite remote sensing data used in this study are publicly available and free of charge. ICESat-2/ATLAS data and ALOS can be obtained at https://www.earthdata.nasa.gov (accessed in November 2021), and Sentinel 1/2 data can be obtained at https://developers.google.com/earth-engine/datasets (accessed in November 2021). Further questioning can point to the corresponding author.
